# Conversion or inflammation?

**DOI:** 10.1192/j.eurpsy.2022.1808

**Published:** 2022-09-01

**Authors:** L. Rodriguez Rodriguez, M.J. Gordillo Montaño, S.V. Boned Torres

**Affiliations:** Hospital Can Misses, Psychiatry, Eivissa, Spain

**Keywords:** conversive disorder, Encephalitis, autoimmune, differential diagnosis

## Abstract

**Introduction:**

Autoimmune encephalitis are inflammatory diseases of the CNS mediated by antibodies that attack neurotransmitter receptors or proteins on the surface of neurons, usually in the limbic system. The clinic is different according to the antineuronal Ac involved.

**Objectives:**

To make a correct differential diagnosis between autoimmune encephalitis and primary psychiatric pathologies that may be similar in symptoms through a complete study of the patient including anamnesis, physical examination, imaging tests, cerebrospinal fluid and serum studies.

**Methods:**

Description of a clinical case. A 31-year-old female patient, with no previous history of interest, was brought to the emergency department for a suspected seizure. The previous days she had presented emotional lability, difficulty in concentration and reading, blurred vision, confusion and hemicranial headache. Two days later she returned to the emergency room for insomnia, dysarthria, difficulty in reading, comprehension, naming, and excessive rumination of her problems. Incoherent and repetitive language. The Emergency service requested to rule out a conversive disorder.

**Results:**

Neuropsychiatric manifestations (anxiety, depression, behavioral disturbances, insomnia, memory deficits, psychomotor agitation, mania, auditory and visual hallucinations, delusions) are the first symptom in 70% of autoimmune encephalitis due to anti-NMDA antibodies and usually respond poorly to psychiatric treatment, making the treatment of the primary cause necessary for the remission of these symptoms.

**Conclusions:**

Given their increasing recognition and prevalence, autoimmune causes should always be taken into account in behavioral changes, cognitive or consciousness impairment of subacute installation, especially in young patients and once infectious, metabolic and vascular causes have been ruled out with an appropriate complementary study.

**Disclosure:**

No significant relationships.

